# Sarcome granulocytique du rachis dorsal: une cause inhabituelle de compression médullaire

**DOI:** 10.11604/pamj.2017.27.56.11935

**Published:** 2017-05-24

**Authors:** Zeineb Alaya, Béchir Achour

**Affiliations:** 1 Service de Rhumatologie, Hôpital Farhat Hached, 4000 Sousse, Tunisie; 2 Service d’Hématologie, Hôpital Farhat Hached, 4000 Sousse, Tunisie

**Keywords:** Sarcome granulocytique, leucémie aiguë myéloblastique, paraplégie, compression médullaire, IRM, Granulocytic sarcoma, acute myeloblastic leukemia, paraplegia, spinal cord compression, MRI

## Image en médecine

Le sarcome granulocytique ou chlorome est une tumeur maligne extra-médullaire rare, composée de cellules myéloïdes immatures. La localisation rachidienne épidurale est exceptionnelle. Nous rapportons le cas d'un patient de 21 ans présentant une paraplégie complète avec un syndrome hémorragique (hématurie et ecchymoses diffuses). L'IRM rachidienne en séquence sagittale T2 (A) et axiale T1 après injection du gadolinium centrée sur D4 (B) a montré un processus expansif épidural postérieur étendu de D4 à D7 de signal hétérogène T2 rehaussé intensément après injection qui comble largement le canal médullaire et exerce un effet de masse sur la moelle dorsale en regard sans signe d'atteinte osseuse. L'hémogramme a montré une pancytopénie. Le frottis sanguin a objectivé 55% de blastes. Le myélogramme a révélé une leucémie aiguë myéloblastique (LAM). La cytologie du liquide céphalo-rachidien n'a pas montré de cellules malignes. Le patient a subi une laminectomie de D4 à D7 et une résection complète de la masse tumorale dont l'examen anatomopathologique a conclu à un sarcome granulocytique du rachis. Le patient a reçu une cure de chimiothérapie systémique selon le protocole des LAM de l'adulte. L'évolution était marquée par l'obtention d'une rémission hématologique et médullaire avec persistance d'un fauchage séquellaire avec un recul de 7 ans.

**Figure 1:**
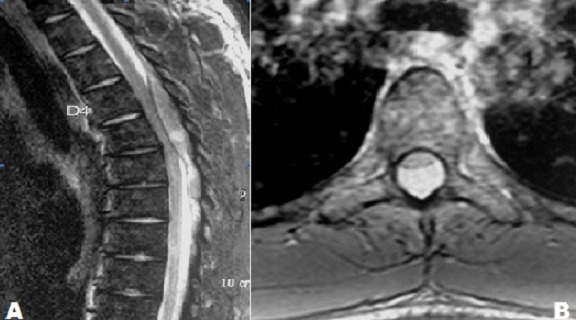
A) IRM rachidienne en séquence sagittale T2 et axiale T1 après injection du gadolinium centrée sur D4; B) processus expansif épidural postérieur étendu de D4 à D7 de signal hétérogène T2 rehaussé intensément après injection qui comble largement le canal médullaire et exerce un effet de masse sur la moelle dorsale en regard sans signe d’atteinte osseuse

